# Draft Genome Sequence of *Rhodobacteraceae* Strain PD-2, an Algicidal Bacterium with a Quorum-Sensing System, Isolated from the Marine Microalga *Prorocentrum donghaiense*

**DOI:** 10.1128/genomeA.01549-14

**Published:** 2015-02-19

**Authors:** Li Zheng, Zhisong Cui, Luyan Xu, Chengjun Sun, Ryan J. Powell, Russell T. Hill

**Affiliations:** a Marine Ecology Research Center, First Institute of Oceanography, State Oceanic Administration of China, Qingdao, People’s Republic of China; b Institute of Marine and Environmental Technology, University of Maryland Center for Environmental Science, Baltimore, Maryland, USA

## Abstract

*Rhodobacteraceae* strain PD-2 was isolated from the marine microalga *Prorocentrum donghaiense*. It has algicidal activity toward its host and could produce *N*-acylhomoserine lactone signals. Here, we present the draft genome of strain PD-2, which contains 5,227,214 bp with an average GC content of 66.19%. There were 4,864 encoding gene sequences and two clusters of *luxI* and *luxR* homologues identified.

## GENOME ANNOUNCEMENT

Marine bacteria associated with microalgae play important roles in the growth of microalgae. They provide nutrients and growth factors that promote the growth of algae (1, 2). However, some algae-associated bacteria that produce alga-lytic compounds have algicidal or algal growth inhibition activities. This is thought to be one of the main factors leading to the succession of certain microalgae populations (3, 4).

*Rhodobacteraceae* is a family of *Alphaproteobacteria* and over two-thirds of the species within the family originate from marine environments. Many of these species are the dominating ones associated with marine organisms like microalga (5), coral (6), and sponge (7). We isolated *Rhodobacteraceae* strain PD-2 from the marine microalga *Prorocentrum donghaiense*. It can produce algicidal compounds that inhibit the growth of its host. This bacterium also synthesizes *N*-acylhomoserine lactone molecules (AHLs) as quorum-sensing signals. At least five AHL compounds were found in the metabolites of strain PD-2 by the thin-layer chromatography (TLC) overlay method using Agrobacterium tumefaciens KYC55 as the reporter. Here, we performed genome sequencing of PD-2 and hope to find a potential relationship between its algicidal activity and quorum-sensing system based on the genomics information.

Genomic DNA of *Rhodobacteraceae* strain PD-2 was prepared as described by Sambrook et al. (8). The genome sequence was sequenced and analyzed by Majorbio Co., Ltd. (Shanghai, China). Genomic DNA was sequenced using Solexa paired-end sequencing on an Illumina MiSeq platform. After trimming, a total of 9,003,358 paired-end reads (500-bp and 3,000 bp libraries) were generated to reach a 391-fold depth of coverage. The reads were assembled using SOAPdenovo version 2.04 (http://soap.genomics.org.cn). The resulting genome sequence consists of 44 contigs (more than 1 kb, *N*_50_ = 386,454 bp) of 5,227,214 bp with an average GC content of 66.19%. There were 4,864 encoding gene sequences with an average size of 904 bp predicted using Glimmer version 3.02 (http://www.cbcb.umd.edu/software/glimmer), giving a coding intensity of 84.1%. All the encoding sequences were annotated by BLAST using the Genes, String, KEGG, GO, COG, and Nr databases. A total of 4,708 (~96.79%) sequences were functionally annotated; 45 tRNA genes for all 20 amino acids and one complete copy of 5S-16S-23S rRNA and two partial copies of 5S and 16S rRNA were identified by tRNAscan-SE version 1.3.1 (http://lowelab.ucsc.edu/tRNAscan-SE/) and Barrnap version 0.4.2 (http://www.vicbioinformatics.com/software.barrnap.shtml), respectively.

From the annotated sequences of *Rhodobacteraceae* strain PD-2, two clusters of *luxI* and *luxR* homologues were identified. Each cluster of the *luxI* and *luxR* genes is contiguous and the *luxR* gene is located in the upstream position. Some scientists also speculated that algicidal activity of bacteria was regulated by quorum sensing, but there is a lack of direct evidence for confirmation on the genetic level (9). The genome sequence of PD-2 may help to understand genetic underpinning of the algicidal activity.

### Nucleotide sequence accession numbers.

The draft genome sequence of *Rhodobacteraceae* strain PD-2 has been deposited at DDBJ/EMBL/GenBank under the accession number AWRV00000000. The version described in this paper is version AWRV02000000.
